# Geographic Atrophy after Reabsorption of Pigment Epithelial Detachment (GARPED) study

**DOI:** 10.1186/s12886-023-02993-3

**Published:** 2023-05-30

**Authors:** Enoch T. Peng, Sean D. Adrean

**Affiliations:** 1 Retina Consultants of Orange County, 301 W. Bastanchury Ave #285, Fullerton, CA 92835 USA; 2 UT Southwestern Medical School, Dallas, TX USA

**Keywords:** Geographic atrophy, Pigment epithelial detachment, Age-related macular degeneration, AMD

## Abstract

**Background:**

To describe the occurrence, rate of geographic atrophy (GA) expansion, and changes in visual acuity (VA) after reabsorption of subfoveal pigment epithelial detachments (PED).

**Methods:**

Included patients had reabsorption of a PED followed by GA. Patients underwent clinical examination with SD-OCT. Images were classified by size with grading occurring post reabsorption. VA was recorded pre-reabsorption, post-reabsorption, and over time.

**Results:**

The average age of the cohort, consisting of 22 eyes from 19 participants, was 86.9 years at reabsorption. Prior to reabsorption, the VA was 20/80 and then declined to 20/200 (*p* = 0.001) with an average follow-up time of 30.2 months. There was no significant VA change after the initial loss with reabsorption. The average initial lesion size of GA was 0.987 mm^2^ with an average growth rate of 0.274 mm/year.

**Conclusions:**

This study longitudinally examined GA growth rate in patients with reabsorbed PEDs. These patients started with a drusenoid or serous PED, had a dramatic reduction in vision and GA that occurred in place of the PED. These GA lesions have a slower growth rate and a smaller area of onset compared to rates previously reported in the literature. They do not show significant VA change after reabsorption. As we have entered the era of GA therapy, these patients may not benefit from current treatments.

## Background

Geographic atrophy (GA) is an advanced stage of age-related macular degeneration and is characterized by the loss of photoreceptors, retinal pigment epithelium, and choriocapillaris [1]. These atrophic lesions generally begin in the perifoveal region and can expand to include the central fovea [1]. The term geographic is used because of the sharp borders due to the loss of retinal pigment epithelium adjacent to unaffected retina resembling continents on a map [1, 2]. The natural history and progression of GA is clinically important to study as it is affecting an estimated 973,000 people in the US and 2.95 million people globally [3]. Geographic atrophy has considerable variability in GA progression rates ranging from 0.53 mm^2^/year to 2.6 mm^2^/year [2]. Smaller lesions typically expand at much slower average rates of 0.74 mm^2^/year until they reach a certain threshold size, typically around 2.5 mm^2^ and then the average expansion occurs more rapidly at around 1.56–1.88 mm^2^/year [2].

Pigment epithelial detachments (PED) are also prevalent features in age-related macular degeneration (AMD) [4]. There are multiple types of PEDs including serous, fibrovascular, and drusenoid [4]. For drusenoid PEDs, the detachment of the retinal pigment epithelium occurs between the RPE basal lamina and Bruch’s membrane and can originate from basal linear drusen deposit accumulation [5]. It is hypothesized that fluid accumulation in Bruch’s membrane occurs from hydrophobic lipid deposition, reducing fluid outflow, and increasing the size of the PED [6, 7]. Similar to drusenoid PEDs, serous PEDs occur between the RPE basal lamina and Bruch’s membrane, however, generally result in larger detachments than drusenoid PEDs [5]. Reabsorption of the PED may occur, causing the photoreceptor outer segments to disappear with a reduction in the number of photoreceptor inner segments, resulting in visual acuity (VA) loss and commencement of GA [8].

As more therapeutic options for GA arrive, the GA that occurs after the reabsorption of PEDs is of potential interest to investigate and further characterize. We looked at a series of patients with subfoveal PEDs that were reabsorbed and examined their initial VA, VA after reabsorption, and VA over time. We also examined the rate of growth of GA over time in this study.

## Methods

This was a retrospective study in which the database of a retina-only practice was searched for patients between April 2007 and July 2020 that had central Geographic Atrophy (GA) following the Reabsorption of a Pigment Epithelial Detachment (RPED), the GARPED study. Local IRB approval was obtained, and we followed all of the tenets of the Declaration of Helsinki. Records of patients that had a subfoveal pigment epithelial detachment (PED) with a size greater than 150 microns in height and 600 microns in diameter that was reabsorbed with resultant GA, were examined. Serous and drusenoid lesions were included in the current study. We excluded patients that had neovascular AMD, did not have a PED that was reabsorbed, or had GA from other causes, including myopic degeneration and those who did not have subfoveal atrophy after PED reabsorption.

The patients in the study underwent a comprehensive ophthalmic examination with Spectral-Domain-Optical Coherence Tomography SD-OCT (Spectralis; Heidelberg Engineering; Franklin, MA) at each visit. VA was recorded prior to PED reabsorption, at the first visit post reabsorption, and then followed over time. Snellen vision was converted to ETDRS vision for purpose of analysis.

SD-OCT images of GA were reviewed and classified by size and progression. Grading of GA occurred for images post pigment epithelial detachment reabsorption using the Heidelberg Eye Explorer program built in software tracing tool to measure the area of the GA, at approximately three-month intervals. Images were read by E.T.P. and reviewed by S.D.A. Square root transformation was performed to determine the linear growth rate. Students paired t-test was performed for statistical analysis.

## Results

There were 855 patients identified in the practice that had a PED from January 2007 to July 2020. 22 eyes from 19 patients met the inclusion criteria. The percent of patients with a subfoveal PED that reabsorbed over the course of the study was 2.2% (19/855). The average age of the cohort was 86.9 at the time of reabsorption (range 58–98 years), which consisted of 73.7% female and 26.3% male patients. In this study the cohort was 84.2% Caucasian (16/19 patients), 5.3% Asian (1/19), 5.3% Hispanic (1/19), and 5.3% (1/19) declined to respond. The percentage of patients with a subfoveal PED that reabsorbed bilaterally was 15.8% (3/19). The average maximum height of the PED, recorded at the visit prior to reabsorption, was 250.2 microns and the average maximum diameter was 1768.6 microns. Of the total eyes, 16 (72.7%) were drusenoid PEDs, 6 (27.3%) were serous PEDs.

**Fig. 1:**
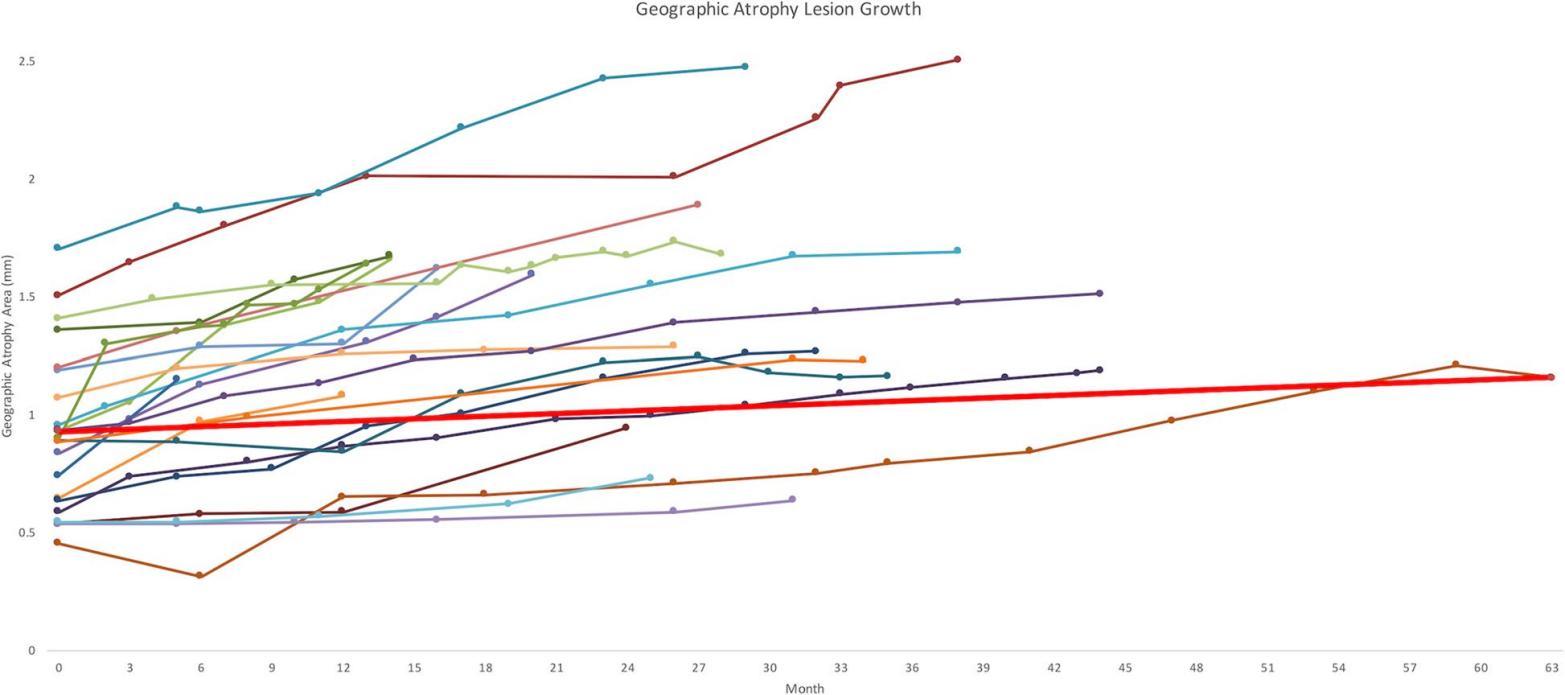
Geographic atrophy lesion expansion following RPED

The average visual acuity (VA) prior to reabsorption was 52.5 ETDRS letters and after reabsorption was 35.2 ETDRS letters, a 17 letter loss, with Snellen equivalents of 20/80 and 20/200 respectively. There was a statistically significant difference in VA just prior to reabsorption and just after reabsorption. (*p* = 0.001) VA at the end of the study was 36.7 ETDRS letters (20/200). There was no statistically significant difference between VA after reabsorption and at the last time point that VA was collected (*p* = 0.725). Seven of 22 eyes [31.8%] lost 1 or less line of vision, while 3 of 22 eyes [13.6%] lost between 1–3 lines of vision, and twelve of 22 eyes [54.5%] lost 3 or more lines of vision after reabsorption of the PED. Drusenoid PEDs lost an average visual acuity of 14.6 ETDRS letters and serous PEDs lost an average of 24.3 letters. There was no significant difference in visual loss between the types of PEDs (*p* = 0.34) or for average VA prior to reabsorption (*p* = 0.18) or post reabsorption (*p* = 0.74). The average range of follow up prior to reabsorption event was 23.7 months with a standard deviation of 26.4 months and a range of 1 to 114 months. The average range of follow up following reabsorption event was 30.2 months with a standard deviation of 12.1 months and a range of 14 to 63 months. The average size of the GA lesion at onset was 0.987 mm^2^ with an average growth rate of 0.274 mm/year after square root transformation (Fig. 1). The average GA growth rates for serous PEDs and drusenoid PEDs were similar at 0.284 mm/year and 0.246 mm/year respectively. No patient developed neovascular age-related macular degeneration (nAMD) during the study period.

## Discussion

Age related macular degeneration is the most common cause of blindness in developed countries and can be broadly categorized into early and late stages [9]. Late stage AMD can be characterized as neovascular AMD involving leakage of new blood vessels, or geographic atrophy involving the loss of central photoreceptors leading to vision loss [9]. AMD progresses to GA from a variety of precursors including PEDs [8]. Studies such as Thiele et. al. found the importance of characterizing specific precursor phenotypes of GA including drusen, refractile drusen, reticular pseudodrusen (RPD), and PED. They found that RPD had the highest risk of progressing to GA [10]. In this study we focused exclusively on further characterizing PEDs, and investigated the growth rate compared to the literature [10].

Drusenoid PEDs are formed from a confluence of soft drusen causing elevation of the RPE which can then collapse and progress to GA [8]. Serous PEDs result in detached photoreceptors due to fluid accumulation which can be accompanied by GA, following serous fluid reabsorption along with a resultant drop in vision [11, 12]. In this study, the patients were examined at 3–6 months intervals prior to the reabsorption event, and typically the event occurred between visits. It was difficult to determine the length of time for the reabsorption event to take place. These PEDs were associated with an increased likelihood of progression to late stage AMD as well as an increased risk of losing more than 15 letters after PED detection [13]. This gives considerable importance to investigating PEDs and their role in relation to progression to GA.

GA is a growing problem as the population ages. It has been extensively studied in multiple randomized clinical trials with a recently approved therapy in February 2023 of pegcetacoplan, an inhibitor of C3 complement. GA may cause minor vision loss at onset if the fovea is spared, however, as it continues to grow in severity and spreads to the fovea, central vision may be lost [14]. Thus, slowing the expansion of GA is important for disease treatment and the growth rate of the lesion is an important indicator of the disease progression [15]. GA has a wide range of growth rates from 0.53 to 2.6 mm^2^/year, spanning a range of more than 2 mm^2^/year, as documented in a literature review[1]. There is evidence suggesting that specific characteristics of GA lesions may predict their enlargement [1]. As a result, it is necessary to better characterize various causes of GA which will prove useful for therapeutic patient selection and in randomized clinical trials.

**Fig. 2:**
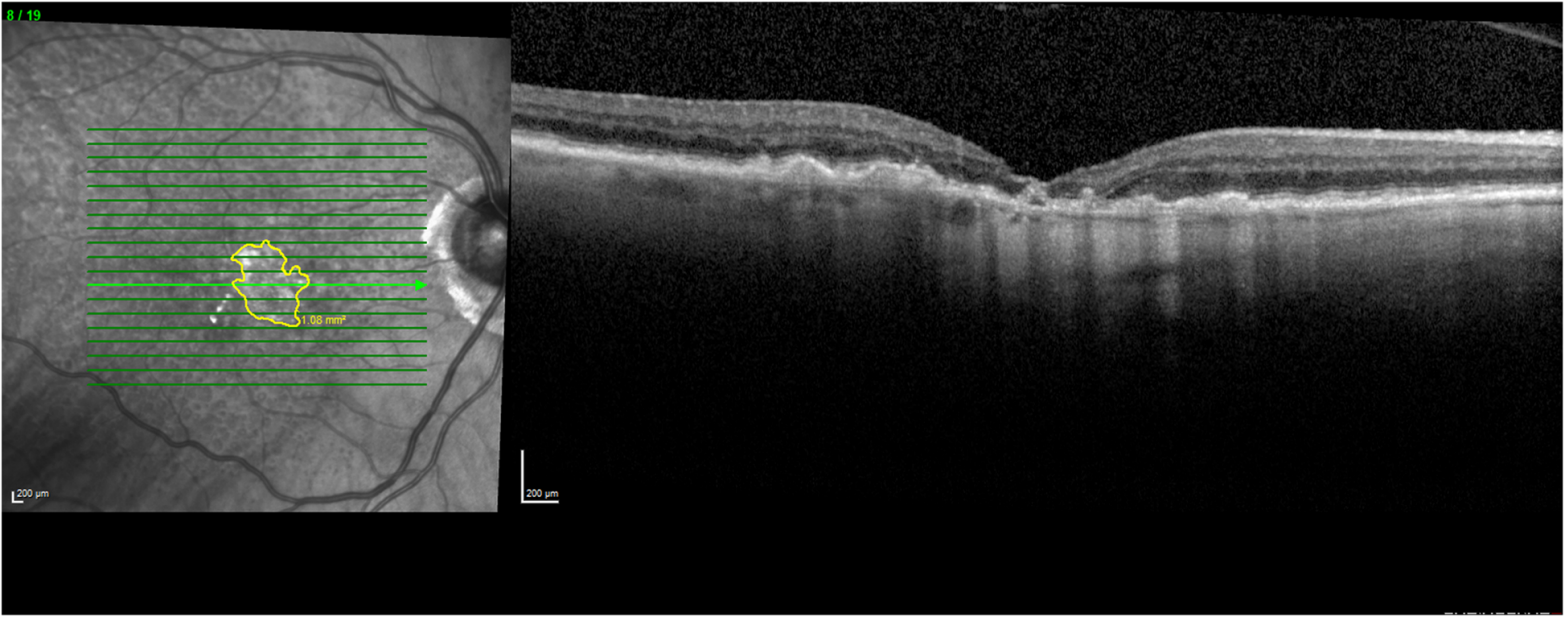
Optical Coherence Tomography of fovea with GA delineation

**Fig. 3:**
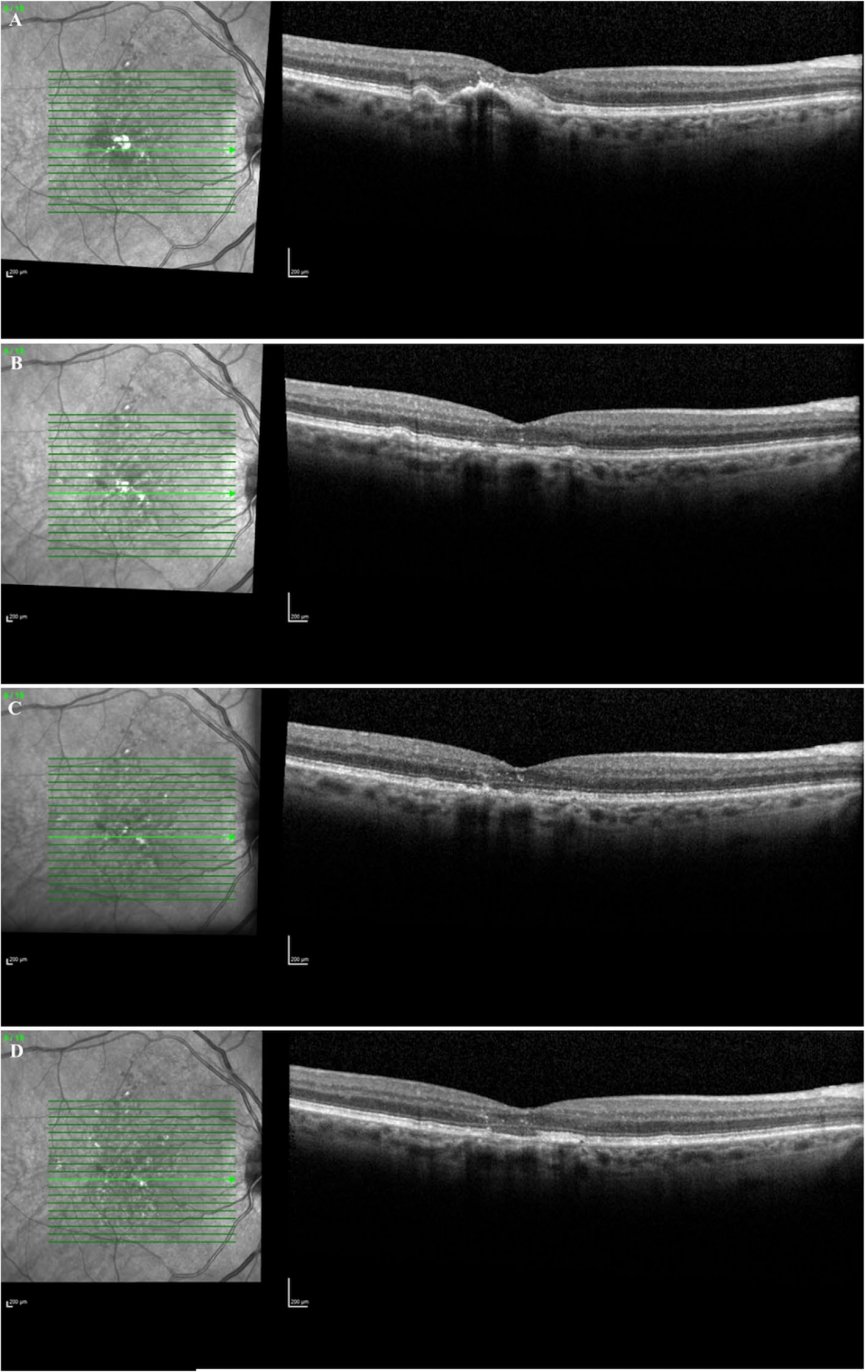
Optical Coherence Tomography of fovea A) Drusenoid PED (20/80); B) Post Drusenoid PED reabsorption 20/200; C) GA growth 6 months post reabsorption (20/100); D) GA growth 12 months post reabsorption (20/100)

**Fig. 4:**
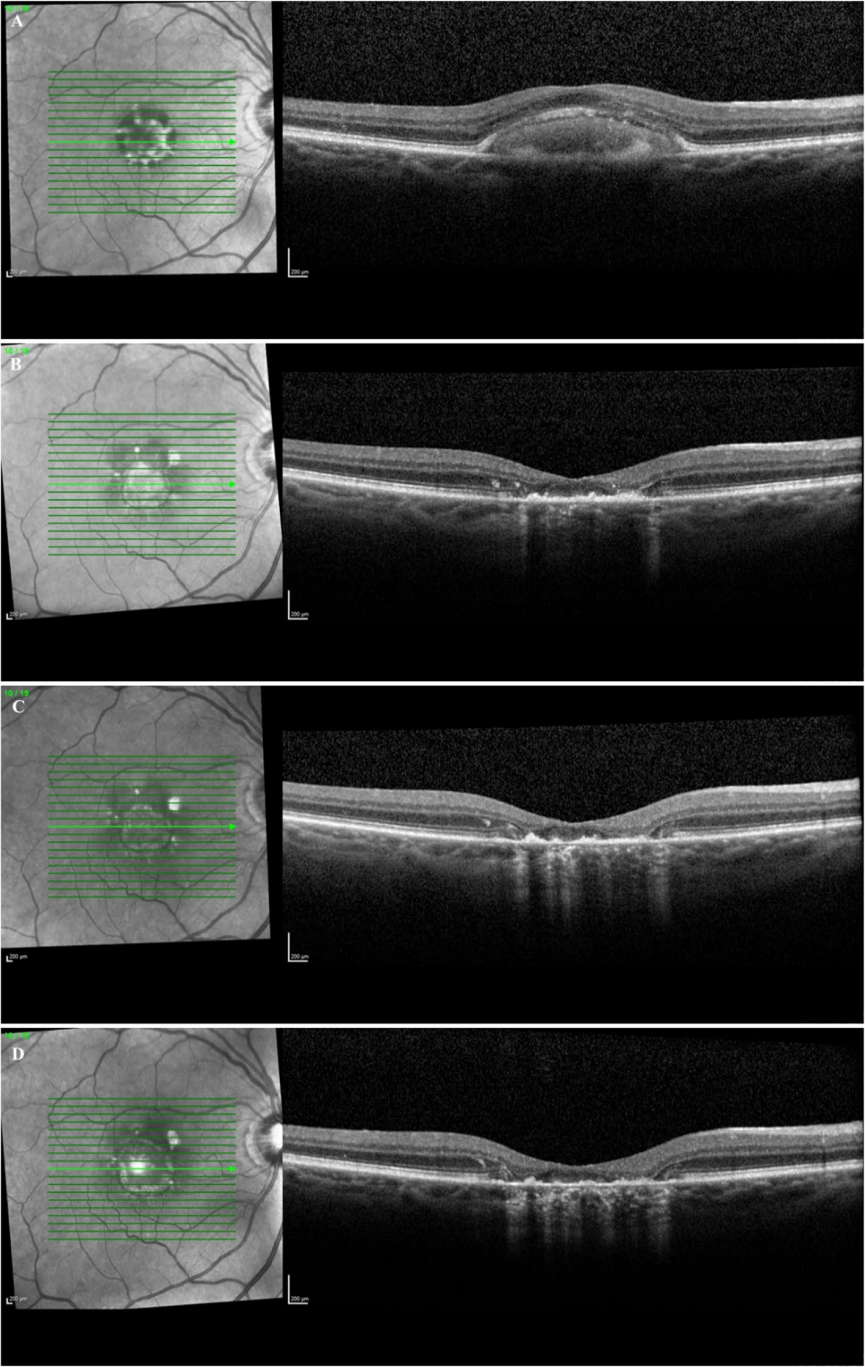
Optical Coherence Tomography of fovea **A**) Serous PED (20/40); **B**) Post Serous PED reabsorption (20/400); **C**) GA growth 7 months post reabsorption (20/400); **D**) GA growth 9 months post reabsorption (CF)

**Fig. 5:**
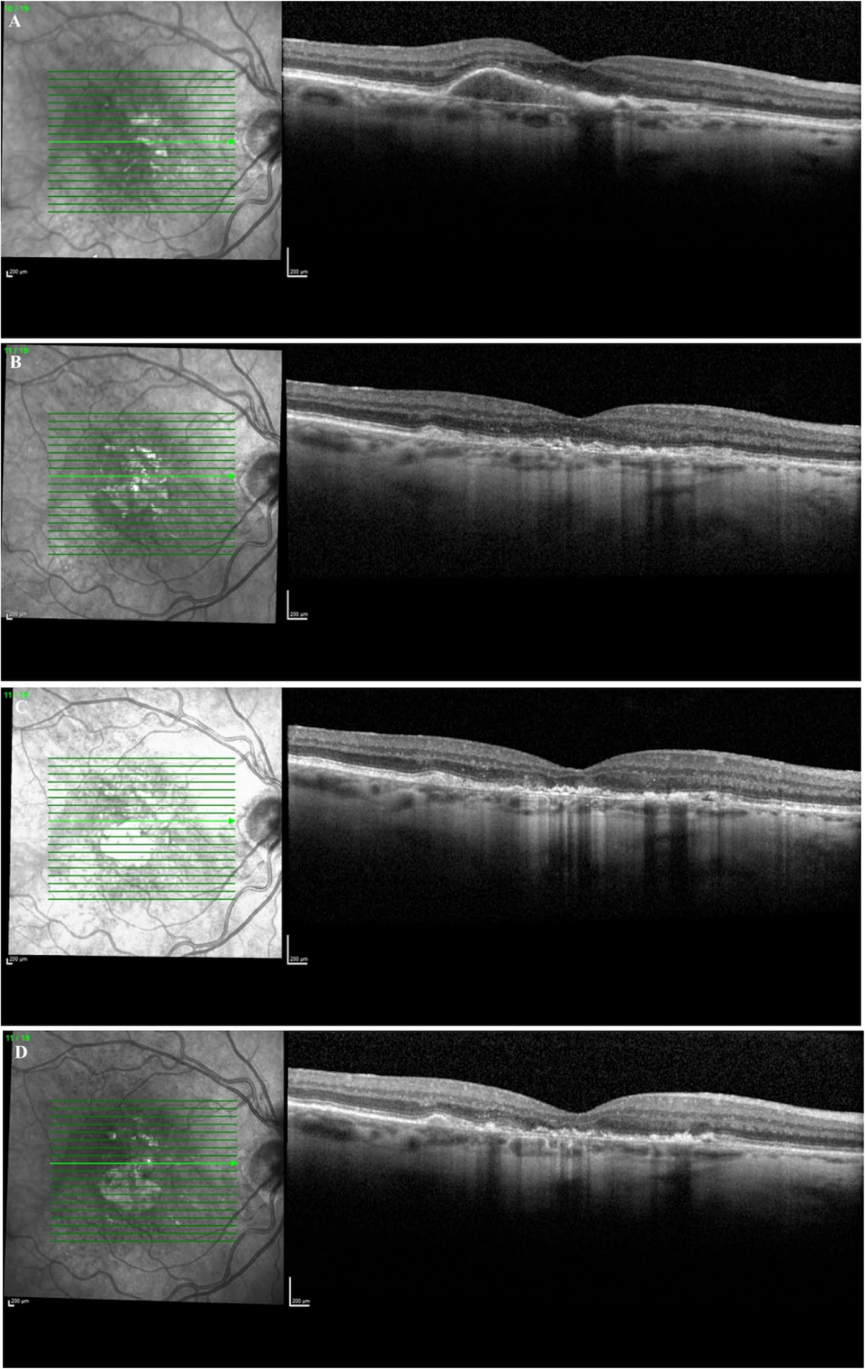
Optical Coherence Tomography of fovea **A**) Serous PED (20/40); **B**) Post Serous PED (20/100); **C**) GA growth 8 months post reabsorption (20/200); **D**) GA growth 12 months post reabsorption (20/200)

In this study, we examined patients that had a reabsorption of their PED with resultant central GA. Not all drusenoid or serous PEDs result in GA. Some may progress to nAMD while others may remain stable with no VA change as seen in larger case series [4]. We observed only 2.2% of patients with PEDs that resulted in reabsorption leading to central GA. In AREDs 28 study, the percentage of drusenoid PEDs that were reabsorbed was 5.4%, while serous PEDs had a wider variation in percent reabsorbed which ranged from 2.5–21% [16–19]. This study only examined those subfoveal PEDs that reabsorbed, leading to central GA (Fig. 2), which likely led to the lower rate of reabsorption noted compared with other studies [16–19]. This typically resulted in a significant vision loss and a central area of subfoveal GA that occurred where the PED existed previously (Fig. 3,4,5). In this study, 54.5% of patients lost 3 or more lines of vision after the reabsorption occurrence (Table 1). Drusenoid PEDs lost an average visual acuity of 14.6 ETDRS letters and serous PEDs lost an average of 24.3 letters. This difference between PED types was not statistically significant, however, this may be due to the small sample size. This has been observed in other studies as well. Marco et al. found a significant correlation between PED reabsorption and resulting GA along with a significant decrease in VA for eyes with a PED reabsorption [20]. Following reabsorption of PED however, the VA in our study group remained constant with no significant change which was also seen in prior studies [12].

**Table 1:** Visual Acuity (VA) and Geographic Atrophy (GA) initial lesion size and growth rate. ETDRS = Early Treatment Diabetic Retinopathy Study

Eye	Initial Lesion Area (mm^2^)	Lesion Growth Rate (mm^2^/year)	Lesion Growth Rate (mm/year)	VA Prior to Reabsorption (ETDRS Letters)	VA Post Reabsorption (ETDRS Letters)	Last VA collected (ETDRS Letters)	Follow up Time (Months)	PED Type
1	0.880	1.512	0.436	70	50	50	8	Serous
2	0.710	0.909	0.360	70	65	65	21	Serous
3	0.920	0.616	0.232	20	20	26	32	Drusenoid
4	0.420	1.389	0.613	61	50	20	12	Drusenoid
5	0.410	0.440	0.230	20	5	5	32	Drusenoid
6	0.290	0.179	0.120	70	65	65	12	Drusenoid
7	1.860	0.760	0.250	70	20	5	15	Serous
8	0.350	0.292	0.164	61	35	35	40	Drusenoid
9	0.800	0.192	0.093	76	20	35	42	Serous
10	0.210	0.217	0.134	55	35	5	59	Drusenoid
11	1.420	0.915	0.325	61	55	50	16	Drusenoid
12	1.450	0.951	0.307	35	20	23	5	Serous
13	1.990	0.315	0.102	55	20	65	16	Drusenoid
14	0.290	0.045	0.038	85	8	60	17	Drusenoid
15	0.300	0.111	0.086	35	20	20	20	Drusenoid
16	1.160	0.217	0.092	65	61	80	12	Drusenoid
17	0.560	2.094	0.758	50	35	23	7	Drusenoid
18	2.280	1.925	0.479	5	5	5	13	Drusenoid
19	0.820	1.611	0.632	55	40	35	14	Drusenoid
20	0.880	0.387	0.158	50	55	55	44	Drusenoid
21	2.920	1.332	0.318	40	35	50	29	Drusenoid
22	0.790	0.056	0.096	50	40	30	7	Serous
Average	0.987	0.748	0.274	52.5	35.2	36.7	21.5	

We observed that PED reabsorption was followed by small central GA with an average size of 0.987 mm^2^. This is different from extrafoveal GA which showed larger average baseline lesion sizes of 2.39 mm^2^ to 6.0 mm^2^ as well as other studies involving central GA secondary to nAMD with average baseline lesion sizes of 4.59 mm^2^ to 5.8 mm^2^ [21–24]. As opposed to these other studies, in this study, GA secondary to PEDs had smaller initial GA lesions. In the current study, since patients were regularly followed before GA onset, the authors recognize that the baseline GA may be smaller, compared to the literature, due to GA onset observation in a closely observed time frame.

The rate of growth of GA is highly variable and is not well understood. Previous studies observe the growth rate of GA to be from 0.28 mm/year to 0.43 mm/year, which are faster than our observed growth rate of 0.274 mm/year, albeit those studies included patients with larger initial GA lesions [21, 23, 25]. Other studies observe growth rates of GA to be more similar to GARPED yet are still larger with rates of 0.28 mm/year and 0.29 mm/year [21, 22]. The study with the growth rate closest to GARPED only involved patients with central GA within a 36 mm^2^ area around the fovea [21]. The slow GARPED growth rate may be due to several factors, such as having a small area of involvement. Small GA lesions typically slowly expand until they reach 2.5 mm^2^ [1]. Since typical GA typically expands in a centripetal fashion, sparing the central fovea, if the fovea is already involved, the growth rate may not be as rapid [1].

GA secondary to PED reabsorption may result from an alternative mechanism to the complement system that is currently being examined by pharmaceutical trials to inhibit GA growth. One possible hypothesis of GA from reabsorption of PED may involve a subclinical choroidal neovascular membrane (CNV) in the PED undergoing apoptosis causing the entire complex to involute leading to GA. A progressive CNV has been shown to mimic drusenoid RPE elevation and photoreceptor death by apoptosis commonly results from neo-vascularAMD [20, 26]. Another possible mechanism of action leading to reabsorption of the PED may be RPE atrophy. The RPE obtains nutrients from the choriocapillaris and prolonged displacement, especially at the apex, may lead to RPE dysfunction. The PED reabsorption and the subsequent loss of drusenoid material may be from reduced secretion from the RPE, and/or the active removal of drusen constituents by microglia and activated Muller cells [24].

Limitations in the current study include its retrospective nature, relatively small sample size, lack of precise dating of PED resorption even with follow up intervals as short as 3 months. This study did not show a significant difference in visual decline between the serous and drusenoid PED which may be a result of the small sample size.

Since new drug therapies work to slow GA by inhibiting the complement system, they may be ineffective in slowing or preventing GA caused by PEDs that are reabsorbed. Our results found that there was a statistically significant loss of central vision after reabsorption of the PED followed by a small central area of GA. This subset of patients with GARPED may not benefit from current anti-complement pharmaceutical agents. Future studies to further analyze the characteristics of the RPE, outer retinal layers and GA as well as the timing of the reabsorption event would be beneficial.

## Conclusion

To our knowledge, this is the first study to date to longitudinally study the growth rate of GA of reabsorbed PEDs over time. These patients will typically start with a drusenoid or serous PED, have a dramatic reduction in vision as the PED is reabsorbed and an area of GA that occurs where the PED was previously. Interestingly, this GA begins with the onset of a small lesion area and progresses at a slow rate of expansion. As we have entered the era of treatment of GA, these patients with GA following PED reabsorption may not benefit from current treatments aimed at inhibiting the complement system. This is due to the slow growth rate of GA over time in this GARPED study, which may not be mediated by the complement system and is possibly due to a mechanism of action distinct from typical GA progression.

## Data Availability

Data are available on request due to privacy or other restrictions.

## Abbreviations

GA: Geographic atrophy
VA: Visual acuity
PED: Pigment epithelial detachment
AMD: Age-related macular degeneration
GARPED: Geographic atrophy following reabsorption of a pigment epithelial detachment
RPD: Reticular pseudodrusen
CNV: Choroidal neovascular membrane
